# Successful treatment of a persistent type IA endoleak with endoanchors following chimney endovascular aortic repair

**DOI:** 10.1016/j.jvscit.2022.10.018

**Published:** 2022-11-03

**Authors:** Aaron Hayson, Ahmad Hallak, Davis Moon, Samuel Money, Waldemar Charles Sternbergh, Clayton Brinster

**Affiliations:** Section of Vascular/Endovascular Surgery, Department of Surgery, Ochsner Health, New Orleans, LA

**Keywords:** Aortic aneurysm, ch-EVAR, Endoanchors, Endoleak, Type 1a

## Abstract

The chimney endovascular aortic repair technique has become an increasingly used option for the treatment of juxtarenal aortic aneurysms; however, type IA and gutter endoleaks complicate this approach in up to 5.9% of cases. Successful treatment of these leaks is challenging. We report a case of a patient who underwent two-vessel chimney endovascular aortic repair in the treatment of a 5.9-cm juxtarenal aortic aneurysm and developed a type IA endoleak. The endoleak was successfully treated with Heli-FX EndoAnchor placement. Resolution of the endoleak was noted at continued follow-up through 54 months.

A persistent type IA endoleak can develop in up to 5.9% of patients after endovascular aortic aneurysm repair with concomitant parallel visceral, or chimney, stents (chimney endovascular aortic repair [ch-EVAR]).^1^ A variety of strategies, including glue or coil embolization or proximal aortic cuff extension with additional chimney stent placement, have been described in this situation, but none is well-established and each is potentially complex and costly. The average base cost of endoanchors is typically $5385 per case.^2^ Coils have a wide range of costs depending on the respective type used. The average base cost of platinum embolization coils (Cook Medical Inc, Bloomington, IN) is $3936.^3^ The cost of an abdominal aortic cuff depends on the vendor and institutional contracts, but typically ranges roughly from $4612 to $5855.^3^^,^^4^ We describe a novel approach to the treatment of a type IA endoleak after ch-EVAR using Heli-FX EndoAnchors. Patient consent and institutional review board approval were obtained.

## Case report

A 77-year-old woman with a history of multiple abdominal surgeries and chronic obstructive pulmonary disease presented with expansion of a known abdominal aortic aneurysm (AAA) from 48 to 59 mm. Her infrarenal AAA neck measured 2 mm in length. Her iliofemoral arterial access was prohibitively narrow for fenestrated EVAR and her cardiopulmonary status precluded open surgical repair. Ch-EVAR was planned to include proximal main body coverage up to but not including the superior mesenteric artery with parallel stents planned for the right and left renal arteries, respectfully.

**Fig 1:**
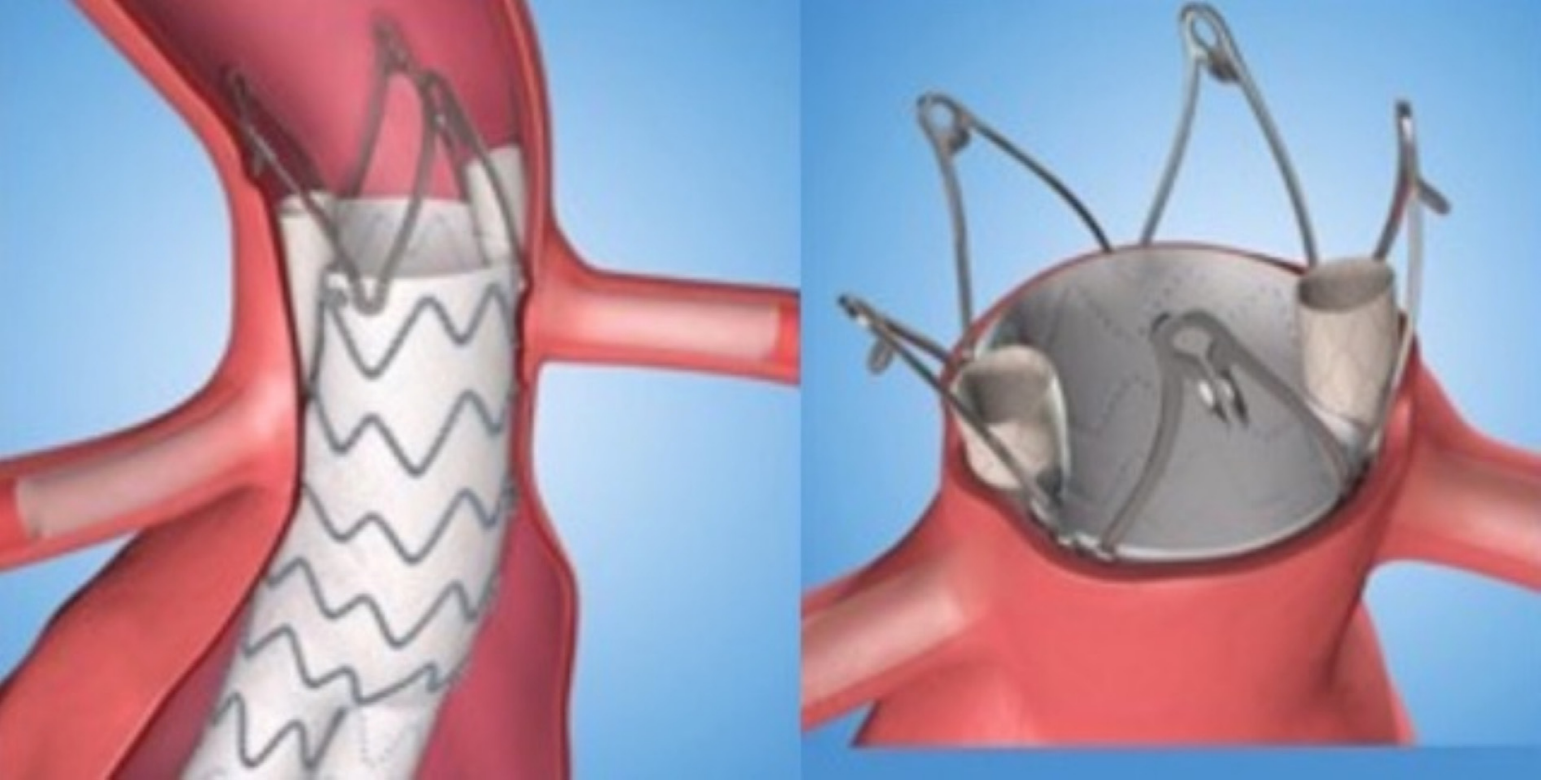
Chimney endovascular aortic repair (ch-EVAR) configuration.

**Fig 2:**
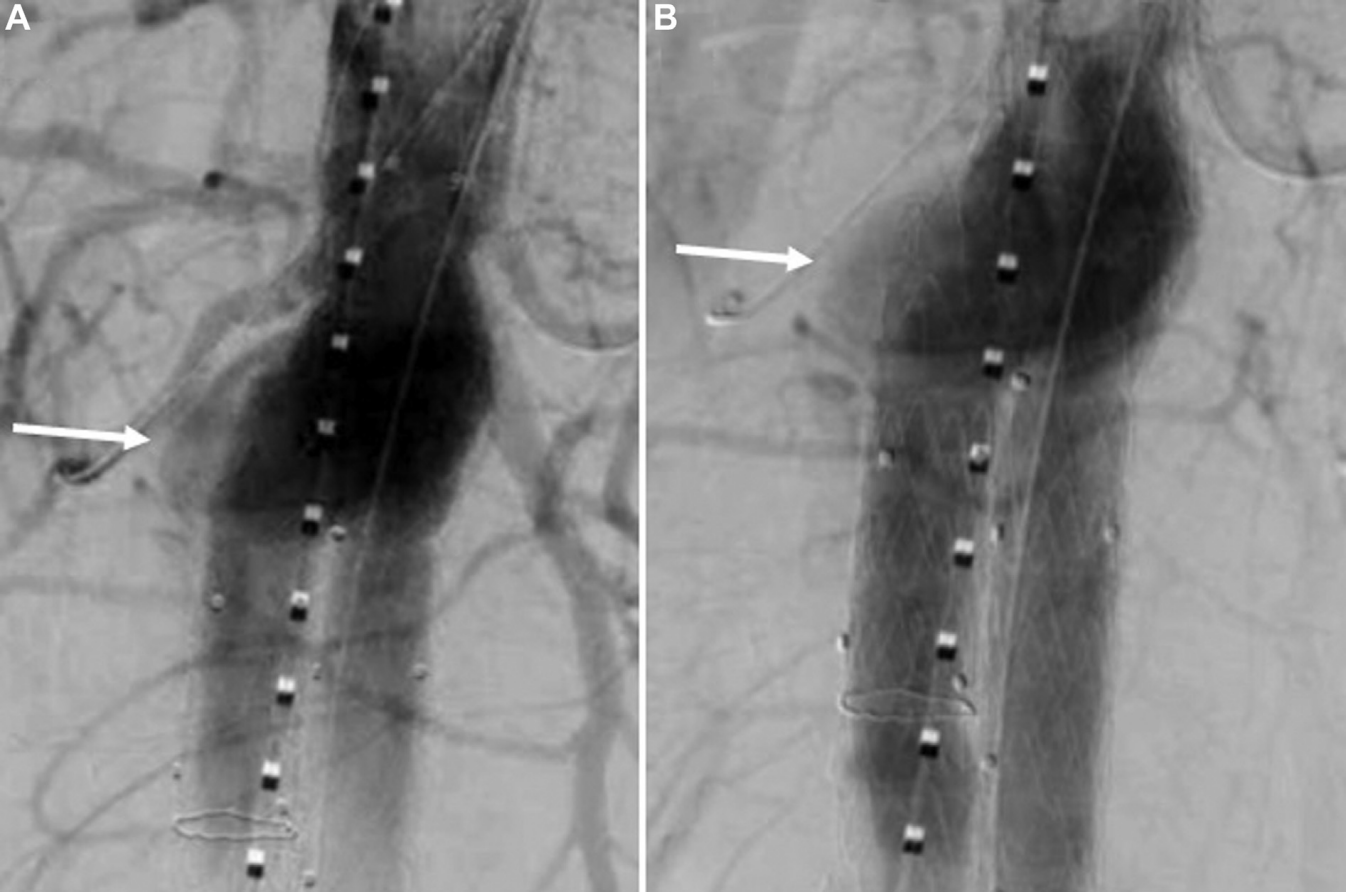
Persistent type IA endoleak after balloon angioplasty during the index operation.

**Fig 3:**
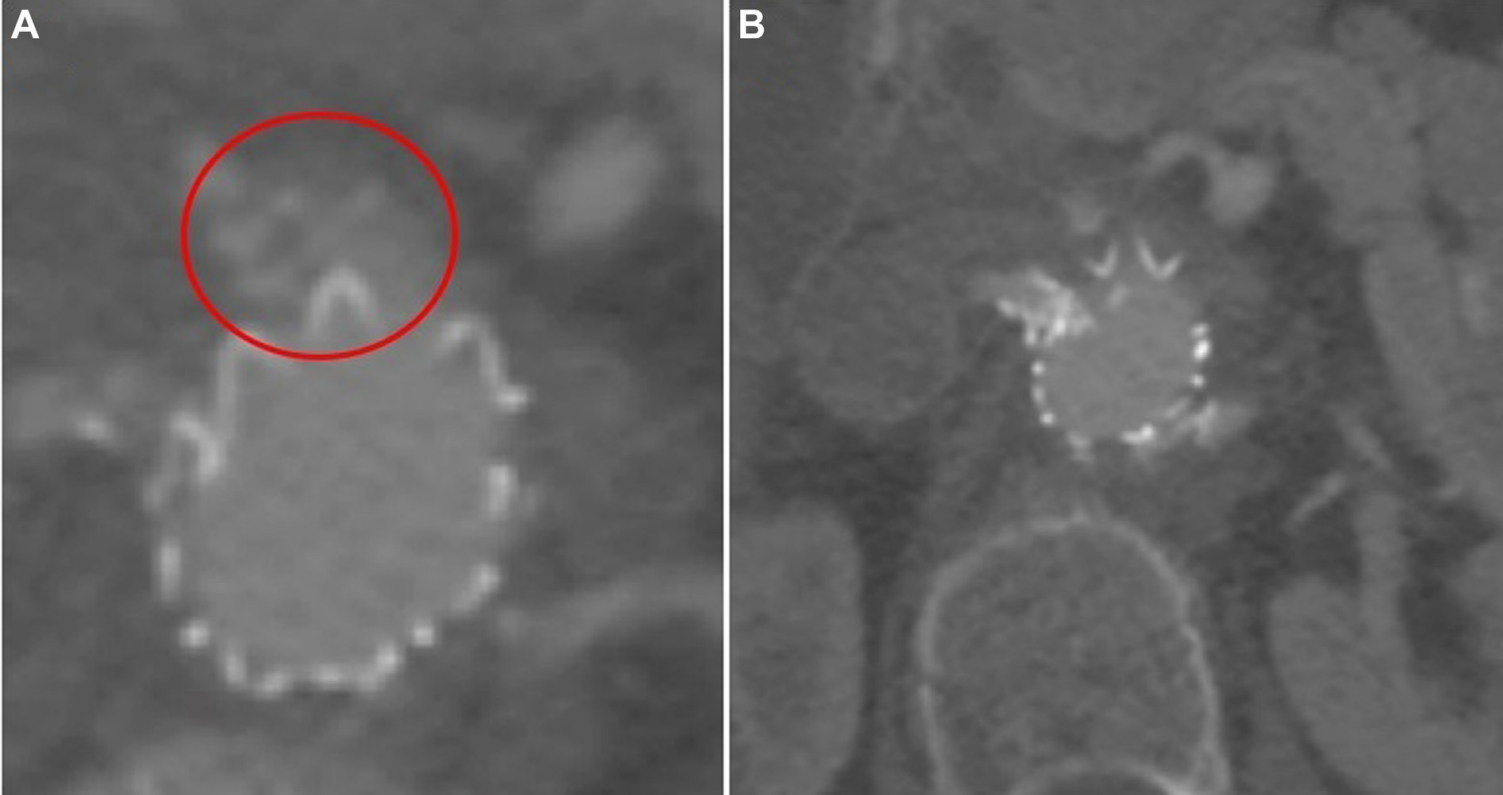
One-month follow-up computed tomography angiogram showed a persistent type IA endoleak.

The proximal aortic seal zone measured approximately 20 mm in length and was uniformly 21 to 22 mm in diameter. A 28-mm main body aortic device was landed just below the superior mesenteric artery. After surgical exposure of the right axillary artery, two 5 × 39-mm VBX (W. L. Gore & Associations, Flagstaff, AZ) stents were deployed in the right and left renal arteries (Fig 1).^5^ A completion angiogram demonstrated a type IA endoleak that persisted after simultaneous proximal aortic and renal artery stent graft balloon angioplasty (Fig 2). The decision was made to observe the endoleak given the documented thrombosis rate of intraoperative type IA and gutter leak during index ch-EVAR.^6^ Computed tomography angiography at 1 month demonstrated a persistent, anterior type IA endoleak (Fig 3).

**Fig 4:**
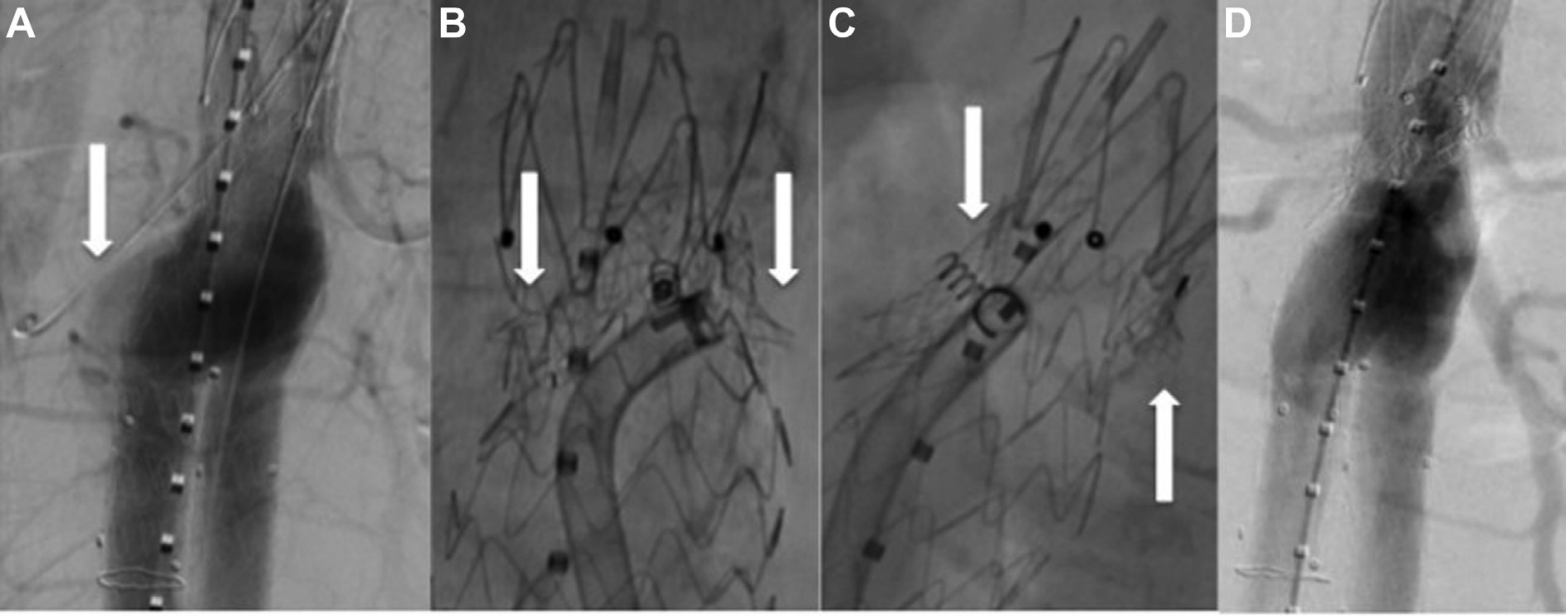
**A,** Type IA endoleak *(arrow)*. **B** and **C,** Endoanchor placement in orthogonal planes (*arrows* renal stents). **D,** Resolution of endoleak.

**Fig 5:**
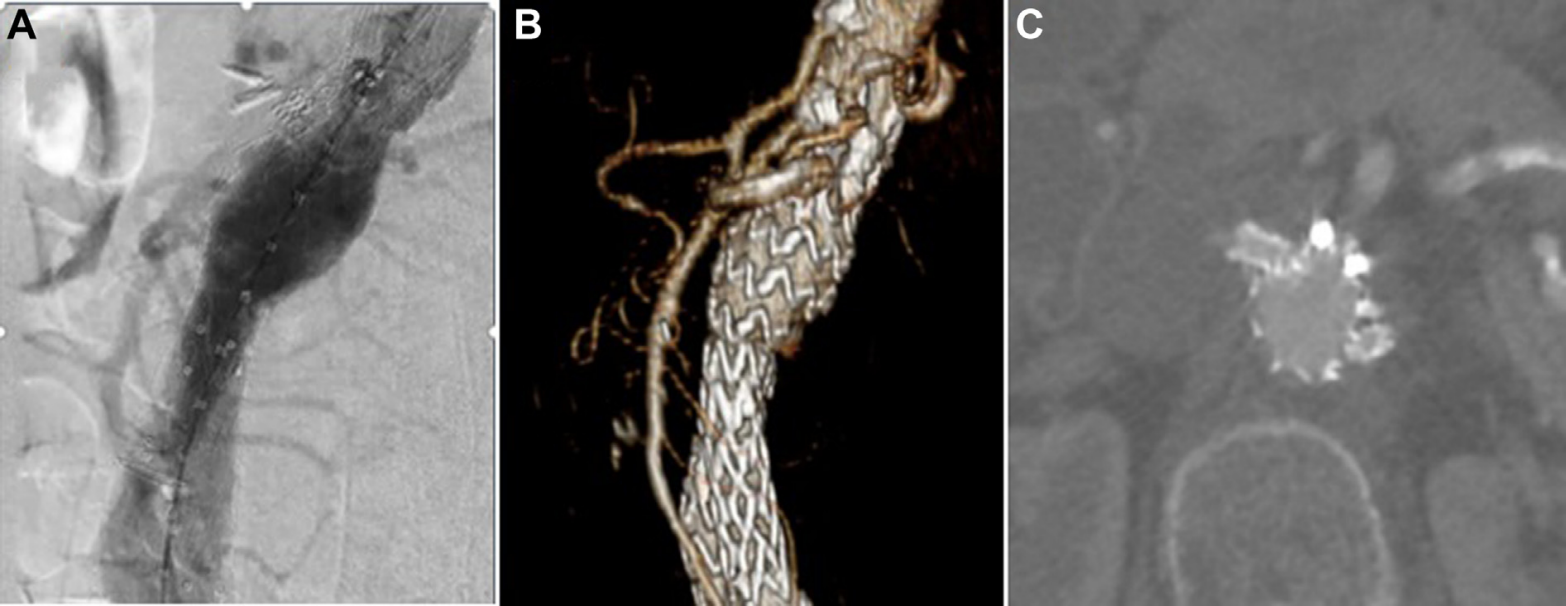
**A,** No endoleak on completion angiogram. **B** and **C,** Follow-up computed tomography angiogram shows renal arteries widely patent.

Reintervention was planned to include Heli-FX EndoAnchor fixation of the proximal, anterior aspect of the main body endograft. A diagnostic aortogram was performed which showed the type IA endoleak at the anterolateral position (Fig 4, *A*). Care was taken during endoanchor delivery to avoid the parallel renal stents using a two-step process: the main body endograft was first engaged by the deployment system, which was then viewed in orthogonal planes before each anchor release to prevent renal stent disruption (Fig 4, *B* and *C*). Five total anchors were deployed and they were localized along the anterior surface of the endograft. These anchors were placed approximately 1 cm below the proximal edge of the endograft and within millimeters of the renal stents, with care taken to remain safely anterior to them. A completion angiogram demonstrated resolution of the endoleak, preserved integrity of the renal stents, and normal target vessel perfusion (Fig 4, *D*). Computed tomography angiography at 12 months showed no type IA endoleak, decreased AAA sac size to 51 mm, and bilateral renal stent patency (Fig 5).

## Discussion

Type IA gutter endoleaks remain a challenge during ch-EVAR, with an incidence of 10.7%.^7^ Various methods have been reported to treat type IA endoleak after ch-EVAR, including embolization with coils, liquid embolic agents, and glue^3^^,^^6^ via multiple anatomic approaches including transarterial, translumbar, transabdominal, and transcaval.^7^ Choi et al^8^ described transarterial and transabdominal approaches using *N*-butyl cyanoacrylate in seven patients, including five type IA endoleaks, one type IB endoleak, and one combined type IA/IB endoleak in which a primary attempt to exclude the type I endoleak failed.^8^ They reported achieving technical success in 86%, with six of seven patients having shrinkage or stability of the aneurysm sac diameter.

The translumbar technique is feasible, but challenging, especially in patients with obesity or cardiopulmonary comorbidities, because patients need to be prone. Massimi et al^9^ reported an effective transcaval approach for a proximal gutter endoleak in a three-vessel ch-EVAR case. Their technique included the use of intravascular ultrasound examination with concomitant biplane fluoroscopy to select the point of maximal inferior vena cava and aortic sac apposition. A transjugular liver biopsy set was used to access the sac via the inferior vena cava with coils delivered along the gutter between the superior mesenteric artery stent and the aortic endograft. Fenestrated and branched endovascular aortic repair (F/BEVAR) also offer solutions in addition to ch-EVAR for these challenging juxtarenal aortic aneurysms. A recent report describing redo F/BEVAR to treat type IA endoleaks after initial F/BEVAR repair has shown promising results.^10^

The use of endoanchors to treat patients with AAA and unfavorable aortic neck anatomy was assessed in the Aneurysm Treatment Using the Heli-FX Aortic Securement System Global Registry (ANCHOR).^11^^,^^12^ However, endoanchors used in combination with ch-EVAR procedures has not been described widely,^13^ although the use of endoanchors in the treatment of juxtarenal AAAs and type IA endoleaks has increased within the last 6 years.^14^^,^^15^ Persistent type IA endoleaks after ch-EVAR are a tough problem. Donas et al^16^ researched gutter-related type IA endoleaks after ch-EVAR. Their conclusions were that, although gutter-related endoleaks were common, they generally resolved spontaneously. Therefore, these endoleaks may be more benign than previously thought. However, a more recent paper by Major et al^17^ showed that a persistent type IA endoleak was associated with a significantly increased likelihood of developing a persistent type IA (*P* < .01) and decreased median survival (*P* < .01), but there was no known aneurysm-related mortality.

We present a unique technique to treat a persistent type IA endoleak after ch-EVAR. A hostile proximal aortic neck is a persistent and challenging problem that possibly increases the risk of type IA endoleak or gutter leak during ch-EVAR. EndoAnchors provide an alternative and potentially more efficient, less expensive approach to achieving fixation in this unique situation. Also, an edge of endoanchor application is that it may prevent aortic dilatation at the proximal seal zone, which could decrease the risk of future aneurysmal degeneration, endoleak, and secondary intervention.^18^ Relative contraindications to the use of EndoAnchors include diffuse aortic thrombus and calcification. EndoAnchor implants should be implanted only into areas of aortic tissue free of calcified plaque or thrombus, or where such pathology is diffuse and less than 2 mm thick.

## Conclusions

Type IA endoleak after ch-EVAR remains a clinical challenge without a reliably effective solution. We present a case of a significant, persistent type IA endoleak after ch-EVAR that was successfully treated with endoanchors. A stepwise deployment process with orthogonal views was used to avoid parallel renal stent damage. Although the use of endoanchors in this situation has been rarely described previously, their selective application may provide a valuable and less complex alternative to other treatment modalities.
